# Digital technologies and healthcare architects' wellbeing in the National Health Service Estate of England during the pandemic

**DOI:** 10.3389/fmedt.2023.1212734

**Published:** 2023-08-02

**Authors:** Evangelia Chrysikou, Eleni Papadonikolaki, Eleftheria Savvopoulou, Eleni Tsiantou, Christian Alexander Klinke

**Affiliations:** ^1^ The Bartlett School of Sustainable Construction, University College London, London, United Kingdom; ^2^ Independent Researcher, Athens, Greece; ^3^ Institute of Education, University College London, London, United Kingdom

**Keywords:** architecture, digital technologies (DTs), healthcare architecture, wellbeing, job satisfaction, National Health Service (NHS), COVID-19

## Abstract

**Introduction:**

UK Built Environment is currently undergoing a digital transformation, as is happening in the National Health Service (NHS) of England. In this paper, the focus was on the intersection of the two sectors and specifically the potential digital transformation of the NHS Estate. The NHS has developed a strategy for its workforce, to improve staff health and wellbeing, and support equality, diversity, inclusion and the development of existing staff. Digital technologies (DTs) can relate to all Estates and Facilities Management priorities, as it cross-cuts all proposed actions. As opposed to most studies on the wellbeing of blue-collar workers, this article focuses on white-collar workers, specifically architects working in the NHS, especially since NHS at this stage is developing two important policies: the New Hospital Programme and the Workforce Action Plan. Therefore, it is important for the NHS to look at the digital transformation strategy in the prism of the other two. As architecture traditionally has low job satisfaction, it negatively impacts wellbeing. This study argues that this might have been accentuated during the pandemic for the architects working in the NHS and dealing with the added pressure from three new major tasks: adjusting the infrastructure capacity to fight Covid-19; and creating the infrastructure for the testing and vaccination programs. DTs in architecture potentially affect job satisfaction in terms of creativity, autonomy, time pressure, organisational commitment, and so on.

**Methodology:**

The methodology comprises a literature review and a pilot of interviews with healthcare architects/designers working in the NHS or on NHS-related projects. The research context is informed by the COVID-19 crisis that brought healthcare architecture to the frontline of the pandemic, with NHS architects creating new wards and vaccination centers, while private healthcare architects designed new hospitals.

**Results:**

In the niche area of healthcare architecture, architects were in their busiest year. Yet, the DTs available to them then could only support limited tasks and did not link well to operational data.

**Discussion:**

To explore how DTs transform the wellbeing of healthcare architects, understanding wellbeing in healthcare architecture in light of digital transformation is crucial for creating the necessary leadership for the sector to grow.

## 1. Introduction

Architecture is the industry shaping the physical context of our urban life. By creating spaces, it is interlinked to societal structures (1). In the case of the healthcare estate, space can have an eco-biopsychosocial impact, affecting the health and wellbeing of staff and patient recovery (2–4). However, even though there are no specific data for healthcare architects, we know that architecture in general has traditionally low job satisfaction and a high prevalence of stressors that negatively impact wellbeing (5). In the prism of digital transformation that is currently happening in the UK built environment, digital technologies (DTs) could support healthcare architects to achieve their aspirations of creating inspiring environments that enhance healing and psychosocial support for patients and staff alike. At the same time, the NHS is undergoing a parallel digital transformation, the transformation of its estate under the New Hospital Programme (6) and the transformation of its workforce (7). Yet, the digital aspects in the New Hospital Programme remain largely undefined and vague and with few and very high-level references in public documents to what actually this digital transformation entails (8). The Estates and Facilities Workforce Action Plan summarize in a table its nine key actions outlined to align to the People Plan themes and will help deliver on these wider NHS priorities. Digital technologies do not appear in that table, even though one could argue that those could be relevant to most items of that table. Reading more closely into the text, “digital development” of staff is explicitly mentioned in the Section 3.9 “Invest in what matters to our people.” From all the potential benefits of digital technologies in the healthcare-built environment industry, this paper specifically highlights the connection to staff health and mental health (8).

We are living in the age of the “Fourth Industrial Revolution,” also known as Industry 4.0. Industry 4.0 is showing advances in communication and connectivity among environments rather than technology itself, triggering a series of innovations such as interactive environments, smart cities, smart materials, 3D printing, virtual reality (VR) environments, and so on, where all driven together draw a radically new world (9). Research on another interactive environment, that of telemedicine, showed the influence of the virtual built environment and its spatial characteristics on the user’s experience during teleconsultations and highlighted the key role of technology in that experience (10). The coming of Industry 4.0 also brings a digital leadership approach, an important factor in successfully adopting digital innovations and technologies and therefore creating competitive advantage (11). The technological innovations of Industry 4.0 involve, among others, the Internet of Things, augmented reality, and sensors. Building information modelling (BIM) also belongs to that group and, in combination with the rest of the technologies, can have spectacular results (12). The concept of digital evolves toward technologies such as BIM and big data, showing that the built environment inclines to solving its problems on the use of technologies relying on big data (13). As seen in the past, architecture could not remain indifferent in such a change of paradigm. The development of new modeling and visualization techniques has opened new ways on how the digital model can be considered, as a shared interface between architecture and the whole sphere of enabling intelligent technologies (9). DTs restructure the nature of work; nevertheless, little fragmented research has been conducted on its relation to job satisfaction, mainly studied in conjunction to job performance (14). Undoubtedly, DTs have affected architectural design with new software liberating forms and creating star-architects. However, it is unclear how DTs impacted job satisfaction.

Our rationale is to explore the impact of DTs in creative industries within the context of healthcare architecture during the largest crisis faced by the NHS—a one-of-a-kind phenomenon. Healthcare architects during COVID-19 generated unique societal value under extreme time pressure to transform entire hospital stocks, double existing bed capacity (e.g., Nightingale hospitals), and provide vaccination space, especially since their job outcomes translated into lives saved. Yet, how did they feel? Healthcare architects during those challenging times and by having to adopt a remote way of working, relied heavily on digital technological tools, either for team working, sharing, communicating, or design, to get the job done. Did they have the right tools for this extraordinary task? It is imperative to understand how DTs affect healthcare architects’ relationship to their work to promote our broader understanding in creative industries. Our overarching/long-term goal is to empower healthcare architects in industry and the NHS—the world’s third largest employer—and improve job satisfaction, NHS operations, and patient-health outcomes. Through this proof-of-concept study, the main aim was to improve architects’ experiences of digital transformation, and particularly job satisfaction. The research focuses on healthcare architects who experience intense work restructuring, as demonstrated through the COVID-19 pandemic, bringing architects to the forefront of the healthcare crisis, which should have increased their sense of purpose. The objectives in this position paper were: (1) to understand how DTs transform work and job satisfaction in architects, e.g., creativity, changes in time and activities, organizational commitment, and career prospects; (2) to identify differences in job satisfaction based on the use of DT between architects in public—facing NHS capital, estates, and facilities offices as opposed to private offices; and (3) to develop and propose actionable insights for increasing job satisfaction in healthcare architecture practices using DTs to transform management practices.

## 2. Job satisfaction and wellbeing in the architectural industry

In a rapidly changing working world, digitalization has come to play an increasingly prominent key role in almost every branch and sector across all industries. The changes that have emerged from the advanced usage of digital tools are immense, and their impact is likely to increase during the decades to come. Still, human beings work with and within a globally increasing digital working environment in which productivity and work outcomes are highly related to the emotional stability and the emotional wellbeing of workers and employees. In conclusion, employees’ attitudes toward their job performance significantly influence their job satisfaction and work-life quality (15).

Job satisfaction can derive from many aspects of the work environment, such as salary, security, office environment, work tasks, career advancement, recognition of work, and the organization’s culture (16, 17). In the architectural industry, job safety and security are significant aspects for an architect’s job satisfaction as well as a healthy work environment, which involves inspiring work projects, personal safety, medical care benefits, organizational wellbeing, opportunities to use their skills in work, and committed teammates (17). Long hours, low salaries, workloads, lack of management and HR support in smaller practices, lack of union, and the need to constantly defend their designs are some of the elements that can negatively influence architects’ wellbeing and job satisfaction (18, 19). The data in the study by Sang et al. support these findings, adding the elements of opportunities for promotion, firm’s management, recognition of work, opportunity to use abilities, variety, and job security (20).

Although the literature tends to focus on blue-collar workers’ poor mental health and high levels of suicide in the construction industry, many studies have explored the mental health and wellbeing of white-collar workers, such as architects and engineers (21). Working in the architectural profession may have a direct impact on a person’s physical and mental wellbeing. Architects are exposed to high levels of stress during work time, and the potential negative consequences of this are reduced performance, harmful coping mechanisms, lower productivity, sickness, or even burnout (21). Even while studying, one in three architectural students in the UK struggle with their mental health, as presented by the Architects’ Journal 2018 Survey (18).

Advancements in digital technologies lead the way for better workplace experiences that allow people to focus more on doing their jobs efficiently. People working in a more digitally enabled workplace have reported a better work-life balance and positive wellbeing in the workplace (22). What about architecture? New technologies related to visual communication have always had an impact on the design process (23). Technology alters the way of thinking and practicing architecture and, simultaneously, architecture demands from technology new tools for conceptualization (24). Starting with the adoption of two-dimensional, then three-dimensional design, moving to BIM and in the near future a greater scale adaptation of the Internet of Things, the profession has come to experience a period of important change that allows architects to create buildings and transform cities that could not be created before (25). This digital transformation has transformed the way architects work, the way practices are run, and the way projects are delivered. Projects are more efficient, and all members of the design team can work together better, along with the client, as part of a virtual team, saving both money and their time by minimizing on-site changes and post-construction remedial work (26, 27). Errors are reduced and the quality of the architect’s work is improved as he or she is able to communicate with the other parties involved with precision, meaning reduced errors and duration of the design phase and project delivery time. On the other hand, according to a RIBA and Microsoft report (27), there could be some concerns regarding the adoption of digital technologies in the profession, such as whether older generations could embrace the benefits of digital transformation in their work or concerns regarding privacy and security. In addition, although digitalization could offer great opportunities for flexible work in both time and location, it could affect the work-life balance, as it could blur the boundaries between work and life by creating the need to be online constantly, increase the need to multitask while teleworking at home, or give rise to difficulties with psychologically detaching from work during free time (28).

## 3. Digital technologies and architecture in the COVID-19 era

The COVID-19 outbreak has significantly affected the construction sector, with a decline in construction investment, with delays in existing contracts, and, in some cases, with the closure of construction sites (29). According to Channon (30), architects experienced a time of change in every aspect of their daily life, from sleeping patterns to the use of new technologies and social media—which was challenging for many people—having a direct impact on their wellbeing (31). The new working patterns, such as work from home or flexible working, as well as the impending recession, increased the stress of architects (32). Since the beginning of the pandemic, almost 48% of the architects that participated in the third RIBA COVID-19 survey (33) experienced a negative impact on their mental health, an increasing issue within the profession compared to the two previous surveys in May and March 2020. In the UK, in the first quarter of 2021, based on research carried out by Bespoke Careers (34), the percentage of British architects participating in the survey who planned to leave their job increased to 47% from 36% before the pandemic and the percentage that felt appreciated in the UK dropped from 54% at the start of 2020 to 45%. Long hours and the increase of working weekends and evenings since the start of the pandemic was up to 53% in the UK (34). Financial issues due to reduced working hours, long working hours, new working patterns (such as the working from home reality and work from home-schooling pattern), and loneliness were some of the factors architects had to deal with during the pandemic, which affected their well-being and job satisfaction, an uncertainty architects have also faced in previous financial crises, such as during the collapse of Lheman Brothers in 2008 (35). During this period, there was an urgent need for every company, architectural firms included, to adapt to digital methods, such as teleconferencing, cloud-based services, and digital modeling. In the construction industry, there was a noticeable increase in the use of DTs, such as data acquisition, communication of data and information, and data processing (36). Architects shifted toward remote ways of working, relying even more on digital technologies. An interesting factor is that private sector stakeholders perceived this shift as a major incentive in doing things differently, in a more sustainable way to survive and even thrive in the future (29). Architects relied further on digital technologies to work with their colleagues and have virtual meetings with their clients. Remote work tools—virtual, augmented, and mixed reality platforms—are starting to make their appearance in providing a more holistic experience for both architects and clients (37). For example, data communication tools, such as discussion boards, real-time data manipulation, cloud computing, or work sharing, were some of the digital tools that would help architects overcome challenges, such as working closely with colleagues or discussing the project with a client from a long distance (36). Healthcare architects, in particular, both in the public and private sector, are going through an extremely tight period. Due to the limited literature review on how this current situation has affected their wellbeing and the role digital technologies played for them during the pandemic, the authors ran a series of short interviews with healthcare architects.

## 4. Methodology

The methodology used involved a literature search and a small pilot with interviews. The literature search took place between May and November 2021. It involved gray literature, UCL Library Services SFX@UCL, PubMed, Academia.edu, ResearchGate, Emerald Insight, SAGE Journals, and Google Scholar. Indicative keywords included the following: architects and job satisfaction; architects’ wellbeing; NHS admin staff and job satisfaction during Covid-19; digital technologies and architecture; digital technologies and medical architects; Industry 4.0; and architecture. In addition, the research team ran semi-structured interviews with designers/medical architects collecting data on their work during the pandemic. Twenty possible interviewees were approached (6 men, 14 women). Of them, 12 accepted our invitation to participate in the study. The interviews/data collection took place in November 2021. To be more inclusive, the sample of interviewees was across careers and included healthcare designers working both in the NHS as well as in private architectural practices. The interviewees were first approached by email, seeking consent for their involvement. The email included an introduction of the study topic and purposes, with the abstract of the study attached and a questionnaire for them to complete by either responding to the email or booking an interview (in person or virtual). Further, only one of the architects chose the in-person interview, one the virtual interview, and the majority (10 of 12) provided us with written responses. The same questionnaire used in all cases. The questionnaire consisted of 10 open-ended questions separated into the following three categories: work during COVID-19 (4 questions); digital technologies (5 questions); and one generic question on how they see the impact of their work on the recovery and satisfaction of patients. No personal data were collected and the questionnaires were anonymized.

## 5. Findings

From the interviews conducted, the following data were collected. First, the interviewees were asked about their work pattern during the pandemic, and the vast majority (10 of 12, 83.3%) responded that it was remote, with almost half of them making clear that they were referring to the first lockdown; later, when the rules were loosened, they started going to the office a few days a week. Out of 12 interviewees, only two (16.6%) said that they were working in a hybrid way during the whole pandemic, mentioning site visits, meetings with clinical groups, processes that required their physical presence, such as signing papers as well as resolving IT issues and distributing equipment to staff.

In relation to their workload and if that changed during COVID-19, half of the interviewees (6 out of 12) responded that their workload remained the same but two (16.6%) mentioned working longer hours due to being at home and saving time in commuting, and one (8.3%) reported being more productive. Only two (16.6%) out of 12 stated that their workload increased significantly, with one (8.3%) of them explaining that they had more short-term health projects (such as Nightingale hospitals) but slower long-term ones. Another two (16.6%) out of 12 responded that their workload decreased, and the rest did not give a clear answer if they had more or less work as their answers were focused on the description of their work duties.

The focal point of the last two questions of the first category was the visits to healthcare facilities during the pandemic for work and their feelings about that. Of the 12 interviewees, four (33.3%) stated that they did not need to do hospital site visits but the majority (8 out of 12, 66.7%) admitted that they had to visit healthcare buildings during this period as their work projects were related to them, with most of them specifying that they did not enter COVID-19 wards. The second question was related to their feelings about these site visits. Of the 12 interviewees, four did not do any site visits as stated in the previous question, an additional three (25%) interviewees responded that this question was not applicable to them as the facilities they visited were not COVID-19 wards, and one (8.3%) did not respond to that question at all. The remaining four (33.3%) interviewees described how they felt about visiting healthcare facilities for work purposes during the pandemic. More specifically, one out of four (25%, i.e., 8.3% of the total sample) stated that they were not scared as everyone was wearing masks and social distancing was applied. The other three (75%, i.e., 25% of the total sample) admitted having negative feelings and specifically said “we were kind of apprehensive as we had less knowledge and familiarity with the virus […] Those early days, weeks we were avoiding going anyway, only if it was necessary and couldn’t be done virtually,” “do not exaggerate and say scared but cautious, feeling awkward,” and “Initially it was chaotic and quite stressful to be around the activity. Later visits, where staff understood the procedures, were less anxiety inducing.”

Regarding the second part of the questionnaire about digital technologies, the interviewees were asked about their previous experience with digital technologies at work and how that changed during the pandemic. A range of answers was given as each company uses different digital tools; however, of the 12 interviewees, nine (75%) referred in a positive way to virtual meeting platforms, such as MS Teams, Zoom, and Skype, as the big change in the digital technologies that they started using increasingly due to remote work pattern. Of the 12 interviewees, four (33.3%) mentioned the digital technologies they used for design purposes, such as CAD, Revit, and SketchUp, but, as they highlighted, there was no change in that as they used the same technologies before the pandemic. Of the 12 architects, two (16.6%) responded that they extensively used digital technologies without specifying the type; one of them said the digital technologies improved their work and the other stated that they did not change the work pattern. One interviewee mentioned that due to their pre-existing international projects they were using the Microsoft Remote Desktop, so it was not a big change for them; however, during the pandemic, they did all their communication either through digital platforms or email, phone calls, and WhatsApp.

The next question focused on the type of digital technologies architects used at work during the pandemic and some examples, such as Skype, Zoom, CAD, BIM, and Google Docs, were provided to them. Every interviewee mentioned more than one software, with the majority of responses being concentrated on MS Teams (9 out of 12, 75%). In addition, Skype, CAD programs, and BIM were mentioned in eight (66.7%) of the interviewees’ responses. It seems that the communication platforms and design software were the most popular among healthcare architects. The next type of digital technology that received many answers was the sharing platforms, more specifically Google Docs, mentioned by four (33.3%) architects, and One Drive, mentioned by three (25%) architects. Miro Board was used by 3 (25%) of the 12 architects, some of whom mentioned that they used it for the first time “as we couldn’t use a physical wall for pin-ups” and “we used this to hold virtual crits of different design projects in the office.” Of the 12 interviewees, two (16.6%) said they used MICAD, which is an occupancy property database used by the NHS, and Adobe products, such as InDesign, Illustrator, and Photoshop. The least popular digital technologies, mentioned by only one (8.3%) interviewee, were email, Microstation, NBS Chorus, CodeBook, MS Office, share point, Office 365, NHS estates management software, Dropbox, WhatsApp, Microsoft Forms, and Google as a search engine. As for new digital tools that the architects started using because of the remote work pattern, five (41.7%) did not answer, two (16.6%) said that they did not integrate any new digital technologies into their everyday work, and five (41.7%) responded that they used new digital tools and specifically mentioned Miro board (three out of five), Zoom (two out of five), MS Teams (two out of five), and PDFs in web browsers or simply on iPads for sketch markup exchanges (one out of five).

When asked if they experienced any difficulties with the use of digital technologies, a small majority (7 of 12, 58.3%) responded that they did not, but two of those seven interviewees shared that some of their colleagues were not comfortable and experienced more problems switching to new software. Of the 12 interviewees, three (25%) said that the problem they experienced was the broadband speed, which is not a digital technologies issue. Of the 12 architects, one mentioned that the license management for specific software was problematic and another said that “it was a cultural change overnight,” without clarifying whether they found that change difficult due to the use of digital tools.

The next question in the category “Digital technologies” was related to any other work needs that architects might have that are not covered by the existing digital technologies, asking them to also propose new types of digital tools. Of the 12 interviewees, two (16.7%) did not respond to the question and four (33.3%) answered that their work needs were covered by the existing digital tools. Half of the architects (6 out of 12) said that digital technologies could be useful in other parts of their work and provided some interesting ideas about new tools. In particular, they mentioned augmented reality, entirely virtualized workstations, and a new platform for whiteboard drawing at the same time: “It would have been nice if a more successful version was embedded in Zoom,” “an online portal to create a link between user requests and the Team, a database that captures requests and task completion,” “one integrated system to store, organize, and search for information,” and “blockchain as well as web-based software to replace Photoshop, Word, Excel.”

When asked about their wellbeing and job satisfaction and how they were impacted by digital technologies, the respondents tended to believe that they were impacted both positively and negatively, with two (16.6%) out of 12 not giving an overall answer but analyzing both sides. Most architects referred to the advantages and disadvantages of the use of digital technologies due to the pandemic but five (41.7%) said that their job satisfaction increased, and another five (41.7%) said that their wellbeing decreased. As advantages, many architects highlighted the convenience of working from home, budget-saving due to decreased or no need to travel to the office, flexibility in working hours, increased concentration and productivity, as well as more time spent with their family and lunch at home. One interviewee mentioned that their job satisfaction increased as digital technologies helped her avoid meetings in person with colleagues and made them feel safer. On the other side, the participants who concluded they had an overall reduced job satisfaction mentioned slow computers, problems with Internet connection, eye pain due to long screen hours, backache and migraines, slower progress in some projects at the design stage, lack of interaction leading to boredom, fewer opportunities to learn for junior members of the team, and uncertainty.

The last and more generic question intended to identify the extent to which healthcare architects believe that their work has an impact on patient recovery/satisfaction of patients and if that impact could be increased by the use of digital technologies. Of the 12 interviewees, two (16.7%) did not respond to that question. The majority (8 of 12, 66.6%) agreed that their work as healthcare architects has an indirect impact on patient satisfaction and only two (16.7%) did not say anything about the impact of their work; however, one admitted that digital technologies allowed them to work faster for necessary refurbishments during the pandemic and the other said that “technologies will change the way we think of health systems,” which could be translated as an indirect impact on patient satisfaction. Many of the respondents highlighted the fact that this impact could be increased by the use of digital technologies, allowing them to work faster, more efficiently, and better understand the space. In addition, some of the architects proposed specific tools, such as “simulation tools could help to test different design options and improve the design of healthcare facilities,” “newest system, can help you analyze the traffic flow, the occupation rate, and those data can help us make better decisions in the planning and design process,” “DT could be developed to observe, sense, document people’s activities, behavior, and enhance our understanding of how a better match can be developed,” “digital technologies could also help streamline a patient’s journey through a hospital by cutting down processing time, e.g., registration, pharmacy, etc.,” and “algorithmic diagnosis, genomic medicine and robotic automation […] Primary and community care is being changed by massively increased remote consultation and diagnostics and digital complex disease management.” The above findings are summarized in Table 1.

**Table 1:** Digital technologies and healthcare architects’ work during the pandemic.

Data finding from questionnaires: digital technologies and healthcare architects’ work during the pandemic	
A. Work during COVID19	
1. Work pattern during the pandemic	
Hybrid	16.6% (2/12)
Remote	83.3% (10/12)
2. Workload change during the pandemic	
The same	50% (6/12)
Increased	16.6% (2/12)
Decreased	16.6% (2/12)
No clear answer	16.6% (2/12)
3. Visit healthcare facilities for work purposes	
No need	33.3% (4/12)
Yes (but no COVID-related facilities)	66.7% (8/12)
4. If reply yes at Q3: your feelings	
Negative feelings	25% (3/12)
Not scared	8.3% (1/12)
Not applicable	25% (3/12)
No response	8.3% (1/12)
B. Digital technologies	
1. Experience with the use of DTs at work/change during the pandemic	
Increase use of virtual meeting platforms	75% (9/12)
Use of DTs for design purposes	33.3% (4/12)
2. Types of DTs used at work	
MS Teams	75% (9/12)
Skype, CAD, BIM	66.7% (8/12)
Sharing platforms—Google Docs	33.3% (4/12)
Sharing platforms—One drive	25% (3/12)
Miro board	25% (3/12)
MICAD	16.6% (2/12)
Least popular tools (Microstation, Microstation, NBS Chorus & CodeBook, etc.)	8.3% (1/12)
Integration of new DTs in everyday work	41.7% (5/12)
No integration of new DTs in everyday work	16.6% (2/12)
3. Use of DTs at work—difficulties	
No difficulties	58.3% (7/12)
Bad broadband speed	25% (3/12)
Problematic license management for software use	8.3% (1/12)
4. Propose DTs to cover other work-related needs	
Already covered	33.3% (4/12)
No answer	16.7% (2/12)
Provide interesting ideas about new tools	50% (6/12)
5. DTs impact on wellbeing and job satisfaction	
Job satisfaction increased	41.7% (5/12)
Wellbeing decreased	41.7% (5/12)
Impact both positively and negatively	16.6% (2/12)
C. Generic	
1. Impact of your work on patients’ recovery/satisfaction. Could this be increased by the use of DTs?	
Indirect impact on patients’ satisfaction	66.6% (8/12)
No response	16.7% (2/12)

## 6. Discussion

As already stated, architects have a special relation with digital technologies, as they support them in achieving their design aspirations and in designing inspiring healing environments. By using new technologies, skilled architects could feel more satisfied with their jobs as they can process more information in less time, visualize the end result more efficiently, increase productivity, and manage resources more effectively. This fact was also pointed out by the interviews, as the majority of the participants relied on different types of digital tools for communication, sharing of information and design. With the COVID-19 outbreak and, in many cases, the change in the architects’ work pattern from working at the office to working from home, professionals relied even more on digital technologies as there was an increase in the need for digital tools of communication, such as MS Teams, Skype, and Zoom, and sharing platforms, such as Google Drive and OneDrive, so that colleagues and clients could continue to work together as a team, something that was also pointed out in the interviews. An interesting point is that even though the majority of the professionals were covered by the digital tools available to continue their work, meaning sharing, communicating, and design tools such as BIM and CAD, the interviewees seemed willing to take things a step further and introduce into their work technologies such as virtual and augmented reality for real-life experience, the Internet of Things, artificial intelligence software, and virtualized workstations. It seems that the time to implement 4.0 applications in the design process, such as simulation or advanced robotics, is now more mature than ever.

This period was even tighter for healthcare architects. Even though it was evident from the data collected that in many cases the workload remained the same, this was not the case for professionals involved in health projects related to COVID-19, such as COVID-19 hospitals. The demands were higher and time pressing, having to deliver projects fast as per the demand of the ongoing public health strategy of dealing with the pandemic. Digital technologies proved to be a valuable tool during that period by helping architects deal faster and more efficiently with their architectural projects and, as a result, keep up their job satisfaction. Those working on COVID-related projects had to do site visits during this period, a fact that they admitted having some effect on their wellbeing, especially in the beginning as the whole process was chaotic and stressful and they felt awkward. The vast majority of healthcare architects acknowledge the impact their work has on service users’ recovery and satisfaction. Digital technologies can help in this direction by supporting a healthcare architect’s job and allowing them to work faster and in a more efficient way, providing them more time to work on the qualitative characteristics of space and how to design better for users’ needs—another fact reinforcing their job satisfaction. Yet, reality might be different from the potential, as an architect working in the NHS commented: “can see a great potential but mentioned that currently there no strategic vision in here (NHS). For the DTs, no idea, no clue if anybody is doing anything, no communication of any digital element or strategy. Everything is reactive.”

## 7. Implications

With this paper, the authors tried to explore the relationship between digital technologies and the architectural profession and the role they played for healthcare architects during the COVID-19 pandemic. Due to limited references, a small pilot was run with invited healthcare architects/designers as interviewees. Due to the ongoing public health challenges and the fast way in which healthcare needs to evolve so as to respond to those challenges, the focus on the healthcare architects’ profession is more timely than ever. Further research on a larger scale—with a bigger sample from NHS Trusts and UK architectural practices—or by comparing data collected within different cities in the UK would give a better insight into the matter. In addition, the research could be implemented in the wider architecture industry as it is a leading profession in the use of innovative digital technologies and at the same time one of the industries with a relatively low job satisfaction among its employees. Suggestions for necessary adjustments could be beneficial to the industry.

## 8. Conclusions

The purpose of this study was to unpack the relationship between digital technologies and wellbeing in architecture.

Working in the architectural industry could have a direct impact on a person’s physical and mental wellbeing. As already stated, architecture has a relatively low job satisfaction among its employees. Digital advancements and the impact digital technologies have on architectural design could help in altering this fact. Digital technologies used to and will continue to provide architects with innovative digital tools and software, such as BIM, Internet of Things, communication platforms, and augmented reality. This could help increase their productivity and quality of work, and, as a result, their wellbeing at work.

The COVID-19 pandemic could not be left out of the research context, as it significantly affected the architecture industry by provoking delays in existing projects, or even canceling them, and by changing the work model of architects overnight. This shift also affected their job satisfaction. Even though the interviewees had mixed feelings as far as wellbeing and job satisfaction are concerned due to that change, all could recognize both the advantages and disadvantages of this new reality that included a new remote work pattern, being reluctant to clearly indicate if their job satisfaction was increased or decreased. As far as digital technologies are concerned, as derived from both the literature and the interviews, professionals relied even more on digital advancements to get the job done. Digital technology tools were extremely useful for designers, and as stated by many interviewees, even though their work needs were covered by the existing tools, they were keen to explore new digital technologies and ways to implement them in the design process, making it easier not only for them but also for the users to better understand the space.

For healthcare architects, in particular, this was a demanding period as the need for healthcare building adaptations was high. It was not unusual for them to visit healthcare facilities for work purposes, something that made them uneasy, mainly during the first outbreak of the pandemic. It is important to point out that even though this was a challenging period, healthcare professionals who participated in the study strongly believed that their work has an impact on patients and staff, even if that happens indirectly, and digital technologies could enhance this impact by providing them with the new tools to do so.

## Data Availability

The materials used for the literature search in this study are included in the article. The interview datasets are not readily available because we do not have permission to share the data. Further inquiries can be directed to the corresponding author.

## Appendix

### Questionnaire

#### Work during COVID-19

1. Which was your work pattern during the pandemic (on-site, remote, hybrid)?
2. Did your workload change during that period?
3. Was there a need to visit healthcare facilities for work purposes during the pandemic? If yes, what type of facilities (COVID-19 hospitals/other)?
4. If you had to visit COVID-19 clinics, did you come in contact with patients? What were your feelings?

#### Digital technologies

1. What was your experience in previous years with the use of digital technologies at work? Did that change during the pandemic? If yes, how?
2. What type of digital technologies did you use for your work?:
  a. Skype, Zoom or similar
  b. CAD programs or similar
  c. BIM
  d. Google Docs or similar
  e. Other
  f. Did you use any new digital tools?
3. Did you experience any difficulties with the use of digital technologies?
4. Do you have any other work-related needs that could be covered by digital technologies? If yes, what type of digital tools would you propose?
5. Do you think that digital technologies impact your wellbeing and job satisfaction and how?

#### Generic

1. To what extent do you think your work has an impact on patients’ recovery/patients’ satisfaction? Could this impact be increased by the use of DTs?
